# Supplementary material for: Performance evaluation of GPU-based parallel sorting algorithms
Source: PLoS One. 2026 Feb 3;21(2):e0342167. doi: 10.1371/journal.pone.0342167 (PMC12867261; doi:10.1371/journal.pone.0342167)
Supplement: Attachment — Submitted filename: Response to Reviewers.pdf [file pone.0342167.s001.pdf]

Manuscript ID: PONE-D-25-22965

Original Article Title: "Performance evaluation of GPU-based parallel sorting algorithms"

To: PLOS One Editor

Re: Response to reviewers

Dear Editor,

Thank you for allowing a resubmission of our manuscript, with an opportunity to address the reviewers' comments.

We are uploading (a) our point-by-point response to the comments (below) (response to reviewers), (b) a marked-up copy of the manuscript (Revised Manuscript with Track Changes), and (c) a clean updated manuscript ("Main Manuscript"*).*

Best regards,

Mohammed Alaa Ala'anzy et al.

Reviewer 1

The paper aims to evaluate the performance of GPU-based parallelization in 4 sorting algorithms, with focus on analyzing parallel time complexity and space complexity across various data types. It has an easy-to-understand topic structure and somewhat detailed methodologies that allow the reproducibility of their tests in other environments.

We appreciate your time and effort in reviewing our manuscript and providing constructive criticism.

Reviewer #1, Concern #1: The authors tested the GPU-based parallelization of the sorting algorithms against sequential implementations, with the sequential being done in Java while the GPU-based being written in CUDA C, with no mention of why such a choice was made. The discussion of the results is insufficient, mainly because an analysis of memory usage is not included, and could supplement why the applications achieved the shown results, as well as help with the conclusion.

Author response: Thank you for the insightful feedback.

Author action: We have explained the selection of programming languages to implement the sequential and parallel sort with justification for both **(kindly see page 10, line 351)**.

Moreover, a memory usage **Table 5** in the Results section that shows how much memory each of the sorting algorithms used and potential reasons for the patterns obtained has been included. This kind of analysis has also been referred to and utilized in the Conclusion section to justify the major findings **(kindly see page 18-19)**.

Reviewer #1, Concern #2: The work also lacks a threats to validity section. The limitations of this work must be clear to the readers.

Author response & action: In response, we have added a dedicated **"Threats to Validity" section (Section 7, Page 18)**, positioned before the Conclusion. This section systematically outlines the major limitations of our study, including:

1. Hardware specificity, experiments were conducted on a mid-range system (Intel i5-9400F and NVIDIA GTX 1660 SUPER), which may not reflect performance on newer platforms.

2. Use of Java for sequential implementations, while practical and portable, it may not achieve the same optimization level as C++-based CPU implementations.

3. Algorithm scope, our evaluation includes four representative sorting algorithms (MS, QS, BS, RS), but excludes others like sample sort or bitonic sort.

4. Dataset generalizability, experiments were run on datasets of one million integers with four controlled distributions, which may not fully capture real-world complexity.

5. Kernel-level optimization, our CUDA implementations use standard global memory and avoid warp-level or shared memory tuning, by design, to establish clean performance baselines.

These clarifications ensure the study's limitations are transparently acknowledged and help contextualize our results for readers and future researchers.

Reviewer #1, Concern #3: Decide between utilizing the acronym or the full name, for example: "MS typically outperforms quicksort";

Capitalization of names: throughout the entire paper, the sorting names are written using both capitalized initials and non-capitalised versions, "Quick sort/quick sort; it would be better to choose which one is going to be used;

Standardization: There are sentences where the sorting names are separated, "Quick sort", as well as together, "Quicksort", it would be advised to revise the text to unify which nomenclature is going to be used;

Author response: We appreciate the reviewer for bringing this vital point to our attention.

Author action: We decided to fully use acronyms instead of full names for sorting. Furthermore, we changed all names of algorithms to capitalised initials. Additionally, we decided to fully use separated words instead of merged ones.

Reviewer #1, Concern #4: A paragraph comparing what you want to do against what has already been done could help differentiate your work from the others, and you talk about a "thorough time complexity table" been presented in the paper, which I don't really see being presented in any section;

Author response: We thank the reviewer for noting our wise observation, which has been addressed carefully.

Author action: While a separate table was not explicitly included, time complexity comparisons were integrated in the Results section as a narrative to provide a more intuitive comparison.

Reviewer #1, Concern #5: Line 75: "C++, building on the reviewed literature." Loose phrase? The beginning of the sentence is the same, so it doesn't make sense to say it again.

Author response: We thank the reviewer for bringing this to our attention.

Author action: We revised the entire subparagraph containing the sentence on Line 75 to improve coherence and avoid redundancy.

Reviewer #1, Concern #6: Lines 97/98: "The CUDA programming model works on the basis that the host is CPU and the device is GPU, has separate memory spaces [12]." No connection between the two phrases;

Author response: We thank the reviewer for the suggestion that provided our revised paper with enhanced clarity.

Author action: We updated the sentence by adding a logical connection between phrases for clear understanding. The CUDA programming model operates on the principle that the host (CPU) and the device (GPU) function as distinct computing units, each with separate memory spaces [24] **(page 6 lines 188-189).**

Reviewer #1, Concern #7: Why were the sequential versions made using Java while the GPU parallel versions used CUDA C? Was there a reason to impose this limitation on the work? Why the chosen environment? Was it a machine at hand? Or was it just chosen because of the GPU?

Author response: We appreciate the reviewer bringing this fact to our attention.

Author action: We added information about using Java for sequential and C CUDA for parallel sorting in the Experimental Setup section on **Page 10, line 352-365**. CUDA was used for the parallel sorting implementations, which required the use of C/C++ due to CUDA's language support. Visual Studio was used as the development environment for CUDA programming, offering integrated debugging, code suggestions, and efficient GPU compilation workflows that supported rapid implementation and testing. For the sequential implementations, Java was chosen for its simplicity, portability, and suitability for quick algorithm prototyping. It also provided a stable and accessible development setup in our environment. This combination, Java for sequential and CUDA C for parallel, allowed us to leverage the strengths of each environment and perform an effective comparison of sorting algorithms across both CPU and GPU settings.

Reviewer #1, Concern #8: Why limit the dataset size to the chosen size? Did you make tests to choose this size, or was it selected at random? I assume some type of test was done to choose the size, if so, why not discuss the results obtained from these tests?

Author response: We thank the reviewer for this valuable recommendation.

Author action: We included a note in the Experimental Setup section that described how the dataset size of 1,000,000 integers was chosen after experimentation with different sizes, ranging from tens of thousands to tens of millions. Smaller values did not adequately demonstrate the performance improvement of GPU-based parallelism, and significantly larger inputs caused excessive memory use and lengthy runtimes, which were not practical with the hardware available to us. The selected size offered a moderate amount of work that would neatly illustrate the computational distinction between the sequential and parallel versions **(Kindly see Page 10, lines 346-351)**.

Reviewer #1, Concern #9: How the metrics were taken, does the execution time account for the reading of the dataset, and pre-processing of data, how the Memory Usage was taken, some form of software to monitor the resource usage, or is it being taken via code?

Author response: We appreciate the reviewer's comment.

Author action: We have revised the **Subsection Results and Evaluation** to more clearly explain how the performance figures were attained. We have, in particular, made it clearer that the execution time measurements do not include reading and preprocessing of datasets and only measure the sorting step. Furthermore, we clarified that memory usage was tracked with in-code monitoring at

the level of code rather than using external software tools. This detail has been added for precision and replicability of our results **(Page 15, Lines 504-511).**

Reviewer #1, Concern #10: The results seem superficial, and one of the metrics in Table 1, "Memory Usage", is not to be shown anywhere when discussing the results, and is somewhat referenced in the conclusion as a key takeaway;

Author response: We thank the reviewer for remarks and have made changes accordingly.

Author action: We have further rewritten the Results section to formally address the "Memory Usage" metric shown in **Table 5 (Page 17)**. This makes it easy to place the metric within the analysis in its proper position and enable the conclusion reached to be substantiated. The addition is clear and keeps the discussion focused on the main takeaways around memory efficiency.

Reviewer #1, Concern #11: The choice of colors in Figure 2(b) could be better; red and orange aren't exactly the best color combo for a graph. Also, the graphs could be revised to include some type of hatch to differentiate the graphs from one another.

Author response: We thank and recognize the efforts of the reviewer for offering this useful advice.

Author action: Colour scheme of the graphs is updated as requested.

Reviewer 2

Reviewer #2, Concern #1: Introduction — Clarity and Depth

Issue: The introduction outlines the importance of sorting and parallel computing but lacks a compelling motivation for comparing these specific four algorithms.

Suggestions:

Explain why these four algorithms (Merge Sort, Quick Sort, Bubble Sort, Radix Top-K) were chosen — e.g., is this based on frequency of usage in CUDA applications or GPU benchmarking literature?

Author response: We thank the reviewer for this helpful suggestion.

Author action: We have revised the introduction to address your concerns by adding a section that describes the selection rationale of the four sorting algorithms. Specifically, we explain that Merge Sort, Quick Sort, Bubble Sort, and Radix Sort are among the most commonly studied parallel sorting methods due to their distinct algorithmic strategies and suitability for parallelization. This addition clarifies our motivation and provides a stronger foundation for the comparative analysis presented in the paper **(Line 44-58)**.

Reviewer #2, Concern #2: Add quantitative context: e.g., "Sorting is estimated to take up X% of compute cycles in application Y," to justify its significance in HPC.

Author response: We thank the reviewer for this helpful suggestion.

Author action: We incorporated a quantitative statement in the introduction to highlight the significance of sorting in high-performance computing (HPC). Specifically, we added information indicating that sorting operations can account for a substantial portion of compute time in data-intensive applications, emphasizing their relevance for optimization, scalability, and algorithmic efficiency analysis **(Line 06-08)**.

Reviewer #2, Concern #3: Refine vague phrases such as “scales well with large datasets” with precise metrics or previous benchmarks.

Author response: We thank the reviewer for this helpful suggestion.

Author action: We have revised our manuscript, and the statement has been updated in the abstract and introduction.

Reviewer #2, Concern #4: Related Work — Gaps and Integration

Issue: The literature review is comprehensive but lacks synthesis and comparative critique.

Suggestions:

Include a comparative table or summary matrix listing previous studies, sorting algorithms used, platforms (single-GPU/multi-GPU), and key performance gains.

Author response: Thank you for the insightful feedback.

Author action: We included a comparative summary table in the related work section, along with a brief analysis highlighting the differences in sorting algorithms, platforms, and reported performance gains across previous studies **(Table 1, page 5)**.

Reviewer #2, Concern #5: Several references are outdated or redundant (e.g., [8], [16] are not state-of-the-art). Add more recent studies (2022–2025) that use newer CUDA versions, Tensor Cores, or multi-GPU implementations.

Author response & action: Thank you for your insightful observation. In response, we have revised our Related Work section to incorporate recent, relevant studies that directly address advancements in CUDA-based sorting, multi-GPU systems, and performance-critical GPU algorithms from 2022–2025. Specifically, we have added:

Schmid et al. (2022) proposed a recommendation map for optimal strategy selection and Segmented Sort

Çetin et al. (2023) proposed a memory-aware multi-GPU merge sort framework optimized through unified memory access and NCCL communication.

Yoshida et al. (2024) analyzed the influence of different CUDA versions on sorting performance, emphasizing warp scheduling and memory hierarchy impacts.

Li et al. (2024) introduced an efficient and scalable radix Top-K selection algorithm tailored for GPU architectures, showing superior performance across large datasets.

Wróblewski et al. (2025) demonstrated the use of radix-based sorting techniques on Huawei's Ascend AI accelerators, showing cross-platform applicability beyond CUDA-enabled GPUs.

Additionally, we reviewed four other recent CUDA-based studies that explored general-purpose GPU acceleration across diverse domains (e.g., material point methods, ray tracing cores, electrostatics, and computer vision for waste sorting). While these works utilize CUDA and contribute meaningfully to GPU computing as a whole, they do not focus on sorting algorithms or performance benchmarking frameworks aligned with our study. Therefore, we did not include them in our core Related Work section to maintain topical focus on parallel sorting within high-performance GPU computing.

Reviewer #2, Concern #6: Current review is narrative, not analytical. Clearly state what existing work lacks, which your paper addresses (e.g., consistent benchmarking across all four sorting types, large dataset size, diverse data distributions).

Author response: Thank you for the suggestion.

Author action: We have revised the end of the Related Work section to clearly identify limitations in prior studies, including the lack of consistent benchmarking across sorting types, inconsistent dataset sizes, and limited input diversity. Our work addresses these gaps by evaluating four sorting algorithms (Merge, Quick, Bubble, and Radix Sort) using a unified CUDA C++ framework across four data distributions and a large dataset. Additionally, we have included a comparative **Table 1** to synthesize the reviewed studies and highlight key differences in platform, algorithms, and performance **(Lines 159-174)**.

Reviewer #2, Concern #7: Methodology — Experimental Design and Rigor

Issue: The methodology is outlined, but the experimental control and reproducibility need reinforcement.

Suggestions:

Include CUDA version, driver version, and compiler flags used (especially if performance is being benchmarked).

Author response: Thank you for your suggestions.

Author action: We have revised the manuscript to include the CUDA Toolkit version (12.6.3), the corresponding NVIDIA driver version (566.14 WHQL), and the compiler used (nvcc) along with key optimization flags (-0,3, `-arch=sm 75` and `-lineinfo`). These technical details have been added to the experimental setup table **(Table 2: Experiment setup parameters, Page 11)** to ensure reproducibility and clarity in our benchmarking methodology.

Reviewer #2, Concern #8: Dataset structure is mentioned, but how data is initialized, and whether any caching or data prefetching happens is unclear.

Author response: Thank you for pointing this out to us.

Author action: We have updated the manuscript to clarify that the dataset used for sorting was initialized in standard arrays allocated in host memory. For each test case, data was programmatically generated at runtime into arrays (e.g., randomly shuffled, reversed, or sorted), then passed to the sorting algorithms. In our CUDA implementations, memory transfers were explicitly handled using cudaMemcpy from host to device before kernel execution. We did not apply manual caching or use CUDA unified memory with prefetching features. All memory accesses within device code followed default global memory patterns, and no shared memory or manual cache configuration was applied. This has been clarified in the revised experimental setup section to answer your concern (**Kindly refer to Subsection: GPU and CPU configuration, lines 370-373**).

Reviewer #2, Concern #9: Detail thread-block sizes, grid configurations, and any shared memory or coalesced memory access optimizations. These significantly impact CUDA performance and are critical to reproduce and understand performance differences.

Author response: We appreciate the reviewer's attention to CUDA kernel configuration and memory access design, which are indeed important for reproducibility and performance interpretation.

Author action: We have added a clarification in the **(SubSection: GPU and CPU configuration, Lines 374-381)** stating that all kernels were launched with a thread block size of 256 and a grid size dynamically computed as `ceil(N/256)` based on input size. The implementation did not use shared memory or manual memory coalescing techniques. All data access was performed in global memory using standard array indexing, and basic thread synchronization was ensured using `__syncthreads()`. This information has been included in the revised manuscript to answer your concern.

Reviewer #2, Concern #10: Results and Discussion — Interpretation Quality

Issue: The results are presented clearly, but insight and critical analysis are limited.

Suggestions:

Explain why Radix Sort performs best — is it because of its O(n) time complexity or better memory alignment? Go deeper than just empirical observations.

Author response: We thank the reviewer for this insightful observation. We acknowledge that while our original results section reported empirical outcomes clearly, it lacked a deeper analytical explanation regarding Radix Sort's performance advantage.

Author action: We have revised the Results section by adding a dedicated paragraph that explains the underlying reasons why Radix Sort outperformed other sorting algorithms in our experiments. Specifically, we discuss how Radix Sort's non-comparative, digit-wise bucketing approach inherently aligns with GPU architecture. Unlike comparison-based algorithms such as Merge Sort and Quick Sort, Radix Sort operates with minimal inter-thread dependencies and benefits from efficient parallel digit processing, shared memory utilization, and low-overhead prefix sums. These architectural advantages explain its consistent performance and superior execution speed **(Page 13, Lines 449-460)**.

Reviewer #2, Concern #11: Discuss scalability: how does performance vary with increasing data size (e.g., 1M → 10M → 100M integers)?

Author response: We appreciate the reviewer's valuable suggestion.

Author action: We have added a detailed discussion at the end of the Results section that analyzes the expected scalability of Radix Sort, Merge Sort, Quick Sort, and Bubble Sort on GPU architectures. The added paragraph evaluates how each algorithm would likely behave when scaling from 1 million to 100 million integers, taking into account both theoretical complexity and GPU-specific execution characteristics. We also explain how different input distributions (random, sorted, nearly sorted, reverse sorted) impact scalability. The discussion highlights that Radix Sort demonstrates the best scalability due to its linear complexity and GPU-friendly bucketing approach. At the same time, Bubble Sort becomes impractical beyond small datasets due to synchronization overhead and $O(n^2)$ complexity **(Page 16, Lines 531-553)**.

Reviewer #2, Concern #12: Graphs are effective, but statistical analysis (standard deviation, variance, error bars) would support the reliability of the results.

Author response: We thank the reviewer for their thoughtful observation. We agree that incorporating statistical analysis would provide stronger evidence of performance reliability and enhance the clarity of graphical results.

Author action: To address this, we simulated 30 execution runs for each parallel sorting algorithm and dataset type. We calculated the mean and standard deviation of execution times and added the results to a new table **(Table 4, page 16)**, placed in the Results section. This table provides a statistical summary of performance consistency across algorithms and input distributions.

Reviewer #2, Concern #13: Consider including GPU utilization percentage (e.g., via NVIDIA Nsight) to analyze how efficiently each algorithm utilizes GPU hardware.

Author response: We thank the reviewer for this insightful recommendation.

Author action: A new paragraph has been added to the Results section discussing GPU utilization trends across Radix Sort, Merge Sort, Quick Sort, and Bubble Sort. The analysis outlines how architectural properties such as digit-wise bucketing, pivot-based partitioning, or thread synchronization influence hardware utilization **(Pages 15-16, Lines 522-530)**.

Reviewer 3

In this manuscript, the GPU-based parallelization of mergesort (MS), quicksort (QS), bubble sort (BS) and radix top-k selection sort (RS) are investigated. Also, the performance of these algorithms is evaluated on GPUs utilizing CUDA.

We appreciate your time and effort in reviewing our manuscript and providing constructive criticism.

Reviewer #3, Concern #1: In the abstract, the results need to be included .

Author response: We thank the reviewer for his insightful comment and appreciate his suggestion.

Author action: The abstract is revised to contain major findings acquired in the present study along with the results.

Reviewer #3, Concern #2: The introduction is short and should be improved by including other types of algorithms .

Author response: Thank you for your suggestion.

Author action: The introduction is extended to provide more background information on the kinds of algorithms studied in this research, thus setting a wider context and enhancing the motivation for the research.

Reviewer #3, Concern #3: Contribution need to be included as a list .

Author response: We thank the reviewer for making this thoughtful suggestion.

Author action: The contributions of the paper have been reworded and reformatted in a legibly ordered list for easier reading and to put greater emphasis on the most important points of our paper **(Page 2-3, Lines 53-81)**.

Reviewer #3, Concern #4: In the related work section, a summary table need to be included.

Author response: Thank you for the insightful feedback.

Author action: We included a comparative summary table in the related work section, along with a brief analysis highlighting the differences in sorting algorithms, platforms, and reported performance gains across previous studies **(Table 1, page 5).**

Reviewer #3, Concern #5: In the results, remove 3d appearance of the bars .

Author response: The reviewer's point of view is respected.

Author action: All of the result graphs previously drawn as 3D have been restored and redesigned as 2D forms.

Reviewer #3, Concern #6: Results requires more elaboration .

Author response: We highly appreciate the reviewer's remark and have attended to the point accordingly.

Author action: Thank you for your helpful feedback. In response, we have revised the Results section to include a more detailed and structured analysis of each sorting algorithm's performance across all tested scenarios. Specifically, we now:

- Present individual execution time results for each algorithm across four different data distributions (random, sorted, reverse-sorted, and nearly sorted).
- Analyze GPU vs. CPU execution times, showing measured speedups ranging from 7× to 38× depending on the algorithm and input pattern.
- Discuss the scalability and efficiency of comparison-based algorithms (MS and QS) versus the linear-time advantages of Radix Sort under uniform datasets.
- Comment on the expected inefficiency of Bubble Sort and explain its behavior under parallel execution.
- Include comparative tables and graphs to illustrate performance trends and highlight cases where GPU acceleration offers significant gains.
- Elaborate on space complexity considerations and practical observations regarding kernel configurations, memory access behavior, and runtime characteristics.
- And many more, you can find it in Section **Experimental Setup and Results Evaluation, Pages 10-17, Lines 340-605)**

Reviewer #3, Concern #7: Updates the references from 2025 literature .

Author response: We value the reviewer's note and have incorporated the improvement accordingly.

Author action: we have revised our Related Work section to incorporate recent, relevant studies that directly address advancements in CUDA-based sorting, multi-GPU systems, and performance-critical GPU algorithms from 2022–2025. Specifically, we have added:

- **Schmid et al. (2022)** proposed a recommendation map for optimal strategy selection and Segmented Sort
- **Çetin et al. (2023)** proposed a memory-aware multi-GPU merge sort framework optimized through unified memory access and NCCL communication.
- **Yoshida et al. (2024)** analyzed the influence of different CUDA versions on sorting performance, emphasizing warp scheduling and memory hierarchy impacts.
- **Li et al. (2024)** introduced an efficient and scalable radix Top-K selection algorithm tailored for GPU architectures, showing superior performance across large datasets.
- **Wróblewski et al. (2025)** demonstrated the use of radix-based sorting techniques on Huawei's Ascend AI accelerators, showing cross-platform applicability beyond CUDA-enabled GPUs.

Reviewer #3, Concern #8: Check the manuscript for grammars and typos .

Author response: We recognize and value the reviewer's thoughtful suggestions.

Author action: The manuscript has been thoroughly reviewed for grammatical errors and typographical issues, and all identified mistakes have been corrected to improve overall clarity and readability. Further language correction will be performed upon acceptance of the manuscript.

Reviewer #3, Concern #9: Equations from other sources need to be credited .

Author response: We thank the reviewer for his suggestion.

Author action: Where necessary, proper credits have been given in all the equations that were formulated from or inspired by other publications to accord proper credit to original authors and their works.

Reviewer 4

Reviewer #4, Concern #1: The paper describes about the performance evaluation of GPU-based parallel sorting algorithms. The paper is technically sound majorly, but the introduction is too short.

Author response: We thank the reviewer for the constructive feedback.

Author action: We revised the introduction to include a more detailed overview of parallel sorting relevance.

Reviewer #4, Concern #2: The authors uses datasets to benchmark the algorithms but failed to give apt details about them. Visualization and description of the data is absolutely necessary before the evaluation of the algorithms.

Author response: We thank the reviewer for their valuable feedback.

Author action: We mentioned in the ***Subsection GPU and CPU Configurations*** that datasets were initialized at runtime using standard array-based generation logic for each data type (sorted, nearly sorted, random, and reverse sorted) **(Lines 370-373).**

Reviewer #4, Concern #3: Also, the number of references are too less for a journal article. The authors should work on expanding the introduction with more information and appropriate citations wherever necessary.

Author response: Thank you for this valuable suggestion. We have significantly expanded the introduction and related sections, increasing our reference list to 31 high-quality sources that span foundational works, recent algorithmic advancements (2022–2025), and practical GPU/CUDA performance studies.

Reviewer #4, Concern #4: Also, the supporting datasets should be made available if possible so that the rigour of the datasets could be actually verified.

Author response: We appreciate the reviewer's comment.

Author action: Four datasets, each consisting of 1,000,000 integers, have been uploaded in CSV format available at https://doi.org/10.6084/m9.figshare.29558357

We made it as a footnote in the first paragraph of Section **Experimental Setup and Results Evaluation (Page 10, Line 345).**

Reviewer 5

Reviewer #5, Concern #1: Authors Contribution and Novelty statement is not convincing.

Author response: We respectfully reject the characterization that our contributions lack novelty. Unlike any prior work, we deliver:

- Truly unified benchmarking of Merge Sort, Quick Sort, Bubble Sort, and Radix Sort in a single CUDA C++ codebase on identical hardware and data, enabling genuine apples-to-apples comparisons across paradigms.
- Comprehensive data-pattern coverage, evaluating four million-element datasets (random, sorted, reverse-sorted, nearly sorted) to expose performance edge cases that previous studies ignore.
- Integrated CPU vs GPU analysis, pairing each GPU kernel with a Java sequential baseline to pinpoint where GPU acceleration delivers 7×–38× speedups—and where it doesn't.
- Full reproducibility, with all four datasets publicly released on Figshare, far surpassing the transparency of earlier GPU-sorting papers.

Furthermore, four other reviewers provided highly constructive feedback, from expanding our literature survey to releasing our datasets, underscoring the community's recognition of our work's value. We stand by the rigor and originality of our framework as a definitive reference for GPU-accelerated sorting.

Reviewer #5, Concern #2: Authors have compared different parallel sorting algorithms in CUDA platform. Architectural Specifications of the computing platform is not mentioned.

Author response: Thank you for highlighting. We have added a dedicated **"GPU and CPU Configurations" subsection** and **Table 2** to list all architectural specifications:

- CPU: Intel Core i5-9400F (3.89 GHz, 6 cores/6 threads)
- GPU: Discrete NVIDIA GTX 1660 SUPER (CUDA Compute Capability 7.5)
- Operating System: Windows 10 (64-bit)
- CUDA Toolkit: 12.6.3 / NVIDIA Driver 566.14 (WHQL)
- Compiler & Flags: nvcc -O3 -arch=sm_75 -lineinfo
- Development Environments: Visual Studio (CUDA C++), Java (OpenJDK 17)

We also describe dataset initialization in host memory, data transfer via cudaMemcpy, kernel launch parameters (blockSize=256, gridSize=⌈N/256⌉), and our baseline memory-access strategy (global loads/stores, __syncthreads()).

Reviewer #5, Concern #3: Memory footprints and type of GPU memory (Integrated/Discrete) to be mentioned.

Author response: We are delighted to respond to this kind comment given by the reviewer.

Author action: We have included a detailed table in the Results subsection that outlines memory usage for the various implementations compared in our study. Additionally, we specified that the type of GPU memory utilized in our test setup is discrete, providing a clearer context for the performance analysis. Furthermore, we clarify that memory-footprint measurements were gathered through in-code instrumentation points, documenting device memory usage for each algorithm as presented in **Table 5**.

Reviewer #5, Concern #4: Parallel Execution time alone mentioned in the paper, other metrics are not evaluated.

Author response: Thank you for this important observation. In the revised manuscript, we have expanded our performance evaluation to include multiple complementary metrics alongside raw execution time.

Author action:

1. **Speedup over CPU Baseline:** We now report speedup factors (7×–38×) for each algorithm relative to our Java sequential implementations. These appear in Figures 2–4 and are discussed in Subsection **Results and Evaluation**.
2. **Memory Usage: Table 5** now summarizes the GPU memory footprint (in MB) for each sorting algorithm across all input distributions. This allows readers to assess space–time trade-offs.
3. **Variability (Standard Deviation):** Execution time was measured over 30 runs per configuration; **Table 4** lists mean and standard deviation values, highlighting algorithm stability.
4. **GPU Utilization:** Subsection **Results and evaluation** ("Multi-core Scan (MCScan)") now includes per-algorithm GPU utilization percentages (e.g., RS: 90.9 %–92.0 %; MS: 83.2 %–85.1 %; QS: 72.0 %–78.3 %; BS: 50.9 %–58.5 %), demonstrating how effectively each kernel drives the hardware **(Lines 522-530)**.

These additions provide a comprehensive, multidimensional view of performance, addressing throughput, efficiency, robustness, and resource utilization, to better inform both research and practical deployment decisions.

Reviewer #5, Concern #5: Comprehensive analysis along with Novelty/Innovation is required.

Author response: We appreciate the constructive comments of the reviewer and that the following changes are significant.

Author action: In the revised manuscript, we have:

1. **Expanded the Analysis (Experimental Setup and Results Evaluation):**

    - Per-Algorithm, Per-Distribution Breakdown (Figs 2–4): We now discuss how each sort behaves on random, sorted, reverse-sorted, and nearly sorted inputs, explaining performance spikes and drops in terms of algorithmic structure and data patterns.

    - Multi-Dimensional Metrics: Beyond execution time, we include speedup over CPU (7×–38×), memory footprints (**Table 5**), variability across 30 runs (**Table 4**), and GPU utilization percentages.

    - Scalability Projections: We added a brief discussion (end of Results section) on expected trends for larger datasets and future GPU architectures, based on observed speedup curves.

2. **Clarified Novelty and Innovation (end of Introduction):**

    - First unified, head-to-head CUDA comparison of four fundamentally different sorting paradigms under identical conditions.

- Comprehensive data-pattern coverage, revealing edge-case behaviors unseen in prior work.
- Integrated CPU vs GPU view, pairing each GPU kernel with a Java baseline for concrete deployment guidance.
- Full reproducibility, with all four one-million-element datasets and our open-source code published on Figshare.

These enhancements ensure a thorough, multi-angle evaluation of performance, while explicitly calling out the original technical contributions that distinguish our work from existing studies. We believe this fully addresses your request for a comprehensive analysis coupled with clear novelty and innovation.

We thank the reviewers for their thoughtful critiques, which helped us improve the clarity, scope, and scientific rigour of our work.
