# Supplementary material for: Performance evaluation of GPU-based parallel sorting algorithms
Source: PLoS One. 2026 Feb 3;21(2):e0342167. doi: 10.1371/journal.pone.0342167 (PMC12867261; doi:10.1371/journal.pone.0342167)
Supplement: Attachment — Submitted filename: response to reviewers..pdf [file pone.0342167.s002.pdf]

Manuscript ID: PONE-D-25-22965R1

Original Article Title: "Performance evaluation of GPU-based parallel sorting algorithms"

To: PLOS One Editor

Re: Response to reviewers

Dear Editor,

Thank you for allowing a resubmission of our manuscript, with an opportunity to address the reviewers' comments.

We are uploading (a) our point-by-point response to the comments (below) (response to reviewers), (b) a marked-up copy of the manuscript (Revised Manuscript with Track Changes), and (c) a clean updated manuscript ("Main Manuscript"*).*

Best regards,

Mohammed Alaa Ala'anzy et al.

Reviewer #1

All my concerns were addressed in this manuscript version.

The authors would like to express their sincere gratitude to the reviewers for their valuable time and effort, which have greatly contributed to enhancing the quality of our manuscript.

Reviewer #1, Concern: There is a strange border on Figure 4.

Author action: The figure has been updated.

Reviewer #2

Thank you for the opportunity to review this manuscript. The study addresses an important and timely research question and is conducted with a clear and methodologically sound approach. The manuscript is well-written, logically structured, and provides sufficient details to ensure reproducibility of results.

The data analysis is appropriate, and the conclusions are well-supported by the results. The literature review is comprehensive and contextualizes the current study within existing work in the field.

I particularly appreciate the clarity in the presentation of figures and tables, and the transparency of the methodology, including data sources and code availability, which align well with PLOS ONE's open science policy.

I have not found any concerns regarding ethical approval, participant consent, or data sharing, and there is no indication of dual publication or plagiarism. The manuscript appears to comply with PLOS ONE's research and publication ethics.

The authors wish to extend their sincere appreciation to the reviewers for their significant contributions of time and effort, which have substantially enhanced the quality of our manuscript.

Reviewer #3:

Reviewer #3, Concern #1: In the abstract, the results need to be included. The results is better to be included in terms of improvement ratio between the presented work and existing works .

Author response: We value the reviewer's note and have incorporated the improvement accordingly.

Author action: In the Abstract section, after comparing CPU and GPU, we added the comparison paragraph on **Page 1** with the most existing works on our topic and our paper on the performance of GPU-based sortings represented as multipliers.

"Earlier GPU-based generations of this type typically achieved acceleration rates between 2× and 9× over scalar CPU code. With newer GPU enhancements, including parallel-aware primitives and radix- or merge-optimized operations, acceleration rates have seen significant improvement. Our experiments indicate that Radix Sort based on GPUs achieves a significant speedup of approximately 50× (sequential: 240.8 ms, parallel: 4.83 ms) on 10 million random sort elements. Quick Sort and

Merge Sort have 97× and 103× speedups, respectively (Quick: 1461.97 ms vs. 15.1 ms; Merge: 2212.33 ms vs. 21.4 ms). Bubble Sort, while significantly improving in parallel (123,321.9 ms to 7377.8 ms for an ≈17× improvement), is considerably worse overall. These experimental findings confirm that the new single-GPU implementations can get speedups ranging from 17× to over 100×, surpassing the typical gains reported in previous generations and comparable to or over rates of acceleration reported for cutting-edge parallel sorting algorithms in recent studies."

Reviewer #3, Concern #2: There are several works that try to improve the algorithm performance by utilizing multi-core, GPU, and multi-threading. The authors need to include some of these work, for example:

[R1] Al-sudani, Ahlam Hanoon, et al. "Multithreading-Based Algorithm for High-Performance Tchebichef Polynomials with Higher Orders." Algorithms 17.9 (2024): 381.

[R2] Hsu, Kuan-Chieh, and Hung-Wei Tseng. "Simultaneous and Heterogenous Multithreading: Exploiting Simultaneous and Heterogeneous Parallelism in Accelerator-Rich Architectures." IEEE Micro 44.4 (2024).

[R3] Mahmmod, Basheera M., et al. "Performance enhancement of high order Hahn polynomials using multithreading." Plos one 18.10 (2023): e0286878.

Author response: We recognize and value the reviewer's thoughtful suggestions.

Author action: We added all suggested papers (R1 - R3) and described their uniqueness and similarities in our topic in the Related Works section on **Page 5, Line 175-187**.

"Apart from GPU-directed approaches, many research works have been done to achieve performance improvement through multithreading and multi-core approaches. As an example, Al-sudani et al. [24] developed a multithreading approach for computing high-order Tchebichef polynomials with significant speed-up in the evaluation of polynomials. Mahmmod et al. [25] achieved significantly reduced execution time for high-order Hahn polynomials through multithreading and achieved remarkable runtime reduction over sequential approaches. At the architectural level, Hsu and Tseng [26] designed a framework for multithreading and heterogeneous simultaneous multithreading in accelerator-rich systems, and showed the effectiveness of utilizing intra-core and inter-accelerator parallelism. These efforts together highlight that multi-threading on CPUs, GPUs, or even heterogeneous accelerators remains an essential way to improve algorithmic performance and is orthogonal to the GPU-specific sorting optimizations explored in this work."

Reviewer #3, Concern #3: Check the manuscript for grammar and typos.

Author response: Thank you for your suggestion.

Author action: The manuscript has been heavily proofread for typographical errors and grammatical faults, and all the errors located have been corrected for overall clarity and readability purposes. Moreover, a comprehensive review by professionals will be performed upon acceptance.

Reviewer 4/5

The manuscript titled "Performance Evaluation of GPU-Based Parallel Sorting Algorithms" provides a well-structured comparison of four classical sorting algorithms—Merge Sort (MS), Quick Sort (QS), Bubble Sort (BS), and Radix Sort (RS)—in both sequential (CPU) and parallel (GPU/CUDA) implementations. The study is clearly written and offers a unified benchmarking framework across four dataset distributions using a consistent hardware setup. The inclusion of execution time, memory usage, GPU utilization, and statistical repeatability across 30 runs contributes positively to the rigor of the experimental section.

We sincerely appreciate the time and effort you have dedicated to reviewing our manuscript. Your valuable feedback and constructive suggestions have been instrumental in helping us improve the clarity and quality of our work.

Reviewer #4, Concern #1: Lack of Novelty: The manuscript does not propose any new algorithms, techniques, or optimization strategies. The selected algorithms are well-established, and their CUDA implementations are widely studied. The work presents confirmatory results rather than offering new insights into algorithmic performance or GPU computing.

Author response: We respectfully acknowledge the reviewer's observation. The scope and purpose of our manuscript were established and accepted as a *performance evaluation* study rather than a proposal of new algorithms or optimization techniques. As clarified in Section 1 (Introduction), the novelty of the work lies in the comparative performance evaluation of four sorting algorithms — Bubble Sort, Radix Sort, Quick Sort, and Merge Sort, using GPU-based CUDA implementation.

The primary contribution of the paper is to provide a detailed empirical assessment of these algorithms under various data conditions (sorted, nearly sorted, random, and reverse-sorted) on modern GPU hardware. This performance evaluation offers a unified experimental framework that can serve as a baseline for future CUDA optimization studies and hybrid algorithmic designs.

We have ensured that the title, Introduction, and Contribution sections clearly highlight this intention and scope, emphasizing that while no new algorithms are introduced, the manuscript contributes by quantitatively validating GPU performance characteristics and identifying potential avenues for optimization on top of the existing implementations.

Reviewer #4, Concern #2: Unfair Baseline Comparison: Sequential implementations are written in Java, while GPU versions are developed in CUDA C++. This introduces a language-level performance bias that undermines the accuracy of GPU–CPU speedup claims. A more rigorous and fair comparison would require both versions to be written in the same low-level language (e.g., C/C++).

Author response: We value the reviewer's note and have incorporated the improvement accordingly.

Author action: We reimplemented all sequential sorting algorithms in C++ to remove the language-level performance bias. The experiments were rerun, and the corresponding figures and graphs were updated to reflect the new C++ baseline results alongside the GPU implementations.

Reviewer #4, Concern #3: Limited Optimization: The GPU implementations do not leverage key CUDA features such as shared memory, warp-level primitives, or memory coalescing. While the authors acknowledge this, it limits the relevance of performance results in a high-performance computing context.

Author response: We appreciate the reviewer's comment.

Author action: We have rerun our parallel implementations with the inclusion of CUDA features highlighted by the reviewer, namely shared memory, warp-level primitives, and memory coalescing. The updated experiments and figures in the manuscript now reflect these optimizations, providing a more accurate representation of GPU performance in a high-performance computing context. **See page 11, line 368**

Reviewer #4, Concern #4: Restricted Generalizability: All experiments were performed on a single GPU (GTX 1660 SUPER) and a mid-tier CPU, without comparison across other hardware platforms. While suitable for baseline analysis, the conclusions should be considered hardware-specific.

Author response: We thank the reviewer for their valuable feedback.

Author action: We have included an acknowledgment in the **Conclusion** section indicating that the experiments were conducted on a fixed hardware setup (Intel i5-9400F CPU and NVIDIA GTX 1660 SUPER GPU). This clarification ensures that readers understand the results are specific to this platform **(page 21, line 731)**. Additionally, we have referenced this in the **Threats to Validity** section as well **(Page 20, lines 681-686)**.

Reviewer #4, Concern #5: Scope of Algorithms: The manuscript focuses on only four sorting algorithms. While these are diverse in paradigm, the exclusion of common GPU-optimized algorithms such as sample sort, bitonic sort, or hybrid strategies limits the comprehensiveness of the study.

Author response: We appreciate the reviewer's perspective; however, we respectfully disagree with the assertion that a performance analysis is required for every algorithm. While we acknowledge the importance of performance evaluation, it's challenging to encompass all studies within a single research article. In our study, we intentionally focused on four representative algorithms: merge sort, quick sort, bubble sort, and radix sort, to establish a clear baseline. As noted in the manuscript, we are aware of this limitation and intend for future research to expand the analysis to include additional GPU-optimized algorithms, such as sample sort, bitonic sort, and hybrid strategies. We aim to incorporate these algorithms in our subsequent studies to enhance the comprehensiveness of our evaluations. **(Refer to page 20, lines 687-691)**

Reviewer #4, comment: Data Availability and Reproducibility: A positive aspect of this work is that the datasets used in the experiments have been made publicly available on Figshare. This supports reproducibility and is commendable.

We truly appreciate your feedback, and we hope our responses help clarify our perspective.

Reviewer 6

Thank you for your time and feedback.

Reviewer #6, Concern #1:

The title of paper is "Performance evaluation of GPU-based parallel sorting algorithms, which is general. Someone expects to see time-efficient recently published algorithms in this research. The following sorting algorithms that are time and complexity efficient new sorting algorithms, especially for parallel realization are missed to be considered in your study and comparisons. In addition to Quick, Merge, Bubble, and Radix sorting algorithms, some new ones, such as Mean-based and threshold-based sorting algorithms for integer and non-integer large scale data sets, Slowsort as a new modified parallel-realization of BitSort for integer data sets, and Clustersort are missed. All of them are based on Divide & Conquer idea to break a big problem to many sub-problems.

- SlowSort: An Enhanced Sorting Algorithm for Large Scale Integer Datasets, Preprint of the accepted paper to be published in Software: Practice and Experience, 2025 (DOI: 10.22541/au.174523890.00820511/v1).

- A Threshold-Based Sorting Algorithm for Dense Wireless Communication Networks, IET Wireless Sensor Systems, Vol. 13, No. 2, pp. 37-47, Jan. 2023 (DOI: 10.1049/wss2.12048). - Cluster Sort: A Novel Hybrid Approach to Efficient In-Place Sorting Using Data Clustering, IEEE Access, Vol. 13, pp. 74359-74374, 2025 (DOI: 10.1109/ACCESS.2025.3564380).

- A General Framework for Sorting Large Data Sets Using Independent Subarrays of Approximately Equal Length, IEEE Access, Vol. 10, pp. 11584-11607, 2022 (DOI: 10.1109/ACCESS.2022.3145981).

- On the Performance of Mean-Based Sort for Large Data Sets, IEEE Access, Vol. 9, pp. 37418-37430, March 2021 (DOI: 10.1109/ACCESS.2021.3063205).

Author response: We are delighted to respond to this kind comment given by the reviewer.

Author action: In the Related Work Section, we mentioned these new papers by explaining the basic mechanism and uniqueness of their approach as a new paragraph (**Page 5, Line 188-201**) because they are close to our topic and we will include them in our future performance analysis.

"New sorting algorithms that emphasize both time complexity and ease of parallel implementation have also been developed in recent years. slowsort, for example, is a generalization of bitSort through an adapted parallel version for sorting large-scale integer data [27], while threshold-based sorting utilizes adaptive thresholds to accelerate tasks in dense wireless communication networks [28]. In the same vein, clusterSort uses the combination of clustering techniques with divide-and-conquer methods to achieve efficient in-place sorting for large data [29]. Additional contributions, such as the independent-subarray model [30], splitting data into balanced subproblems for improved workload distribution, and the mean-based sort algorithm [31], leveraging mean-value guided partitioning for improved scalability, further enrich the collection of modern solutions. Together, these developments provide a clear way forward for sorting research in the direction of algorithms that marry domain-specific solutions to parallel paradigms, and they provide valuable pointers to the extension of comparative studies."

Reviewer #6, Concern #2: In the parallel processing, time and complexity analysis depends on the core with higher time and complexity required, and the difference between different cores in

terms of memory space and time required for processing should be extracted, both mean value and standard deviation. In this case, Mean-based or threshold-based sorting algorithm offer subarrays in parallel realization that are independent of each other up to the end of processing and introduce approximately similar number of elements.

Author response: We thank the reviewer for their valuable feedback. The total runtimes offered here do not always outline per-block metrics. In the real world, however, parallel efficiency is determined by the slowest block or core, and because of this, inspecting workload distribution in mean and standard deviation over processing units is recommended. Heavy variance indicates load imbalance, and blocks complete sooner and wait idle while others finish. However, in our work, such detailed per-block analysis of mean and standard deviation was not necessary for our objectives, since we did not base our work on mean- or threshold-based sorting algorithms. Therefore, we did not perform these measurements. **Table 5 on page 17 presents the execution time statistics for 30 runs of GPU parallel execution.**

Reviewer #6, Concern #3: In Some algorithms, when you finish the processing of one core, its data is sorted and it can be used, but in other algorithms it needs more post processing to find the final sorted data. This case is not analyzed in this research work.

Author response: We recognize and value the reviewer's thoughtful suggestions.

Author action: In the Results section, we implemented a paragraph that fully describes the requirements of each parallel sorting used in our paper. Furthermore, there is an additional table **(Table 7, page 20)** about postprocessing of all mentioned parallel sortings for better understanding (**Page 19, Line 666-675**).

An important parallel sorting algorithm difference is whether the data is globally sorted after execution of the kernel or not. As shown in Table 7, QS, MS, and BS all depend on additional post-processing: QS partition pieces have to be recursively merged into each other, MS requires repeated merging of pairs of subarrays, and BS requires multiple global passes for final ordering. In comparison, RS inherently produces a fully sorted array at the conclusion of the last digit-based pass without any additional merging required. Although our experimental examination was aimed at overall runtime efficiency without isolating these post-processing overheads, it is noteworthy to note this distinction to better understand the real-world efficiency of different parallel sorting algorithms.

Table 7. Post-processing requirements of the evaluated parallel sorting algorithms

Algorithm	Post-processing	Description of required steps
QS	Yes	Locally sorted subarrays must be recursively merged and positioned relative to pivots until globally sorted list is achieved.
MS	Yes	Hierarchical pair-wise merging of pre-sorted subarrays must be repeated until only one fully sorted sequence remains.
BS	Yes	Requires multiple global passes, since local swaps alone do not ensure global ordering.
RS	No	Digit-by-digit passes produce an entirely sorted array as a direct output without additional merging.

Reviewer #6, Concern #4: In addition to compare the elapsed processing time, a complexity order study for time and memory in parallel scenario is needed.

Author response: We thank the reviewer for this insightful observation.

Author action: In the "Conceptual Sorting Algorithms with CUDA" section, we added a new subsection called "Parallel memory complexity" dedicated to the usage of memory in the parallel approach based on previous works that we mentioned. There are basic descriptions of the formulas (Big-O) and references to recent works. (**Page 11, Line 367-381**)

Parallel memory complexity

While parallel time complexity has already been taken into account, the memory requirement of each of the algorithms applied in the GPU-based implementations must also be taken into consideration. Table 2 gives the asymptotic memory complexity of the algorithms being compared in parallel scenarios, including the auxiliary buffers and the recursions' overheads.

Table 2. Parallel memory complexity of evaluated sorting algorithms

Algorithm	Parallel memory complexity (order)
QS	$O(\log n)$ recursion stack + auxiliary partition buffers [40]
MS	$O(n)$ temporary arrays required for merging [41]
BS	$O(1)$ (in-place; negligible additional memory)
RS	$O(n+k)$ for counting/bucket arrays (k = digit groups) [42, 43]

As shown in Table 2, BS is almost in-place, while QS employs logarithmic recursion space and decent buffer space [40]. MS incurs heavy O(n) overhead of auxiliary merging arrays, although modern multi-way variants reduce global memory traffic and remove shared-memory conflicts [41]. RS takes O(n) additional memory along with bucket arrays linear in the number of digit groups, but newer optimizations, such as Onesweep, reduce global memory operations per pass [43], and hybrid radix schemes reduce memory transfers in practice [42]. These memory complexity considerations augment the time analysis and provide a more complete view of algorithm efficiency for GPU-parallel setups.

Reviewer #6, Concern #5: Integer and non-integer data set maybe affect the comparison results, have you considered it in your investigation?

Author response: We thank the reviewer for the constructive feedback.

Author action: In the Experimental Setup and Results Evaluation section, specifically within the Results and Evaluation subsection, **Table 3 (Page 13)** presents the experiment setup parameters. It indicates that we utilized a dataset size of 10,000,000 integers, without incorporating other data types such as doubles. Additionally, the impact of the dataset type was not considered, as it was deemed too limited.

Reviewer #6, Concern #6: In the literature of sorting, we have Worst-case, Medium-case (Moderate-case), and Best-case that depend on how much the data is sorted, originally. It is considered in your work. In contrast, the type of data in terms of the probability distribution is not considered. For example, uniform and Gaussian distributions may force different results.

Author response: We appreciate the reviewer's perspective. However, in our paper, we evaluated the algorithm with nearly sorted, random, and reversed inputs, which represent the conventional best-case, average-case, and worst-case scenarios typically used in sorting literature. These cases adequately capture the primary variations in input order, consistent with standard practices in assessing sorting performance.

While we acknowledge that different probability distributions (such as Gaussian or skewed) may influence performance in specific situations, our research operates under the standard benchmark assumptions where the average case is based on a uniform random distribution. Exploring a distribution-sensitive analysis is indeed an intriguing avenue for future research, but it falls outside the current scope of this paper.

Reviewer #6, Concern #7: When we speak about time complexity order in the level O(n logn), large data sets and very large data sets show the effectiveness of sorting algorithm better than small or medium data sets. In most of references, data sets with 105 and 106 elements are large, and data sets with 107, 108, and 109 elements are very large. Have you considered both of them? Do you have different results?

Author response: We appreciate the reviewer’s comment.

Author action: Tests here are typically carried out using one million (1,000,000)-element datasets, which are typically regarded as "large" in the GPU-sorting world. The reviewer is correct to note that O(n log n) algorithmic trend is more evident at such larger scales, i.e., ten million to one billion elements. To verify this observation further, we conducted long-running experiments on a dataset of ten million (10,000,000) elements. The resulting findings and analysis have been incorporated into the Results section. These larger tests put additional burdens in terms of GPU memory and auxiliary buffers, since scaling merge operations and radix buckets puts larger and larger loads on available device resources. Nevertheless, our testing shows consistent behavior and demonstrates the algorithms' scalability over the range tested.

We thank the reviewers for their thoughtful critiques, which helped us improve the clarity, scope, and scientific rigour of our work.
