# Supplementary material for: Performance evaluation of GPU-based parallel sorting algorithms
Source: PLoS One. 2026 Feb 3;21(2):e0342167. doi: 10.1371/journal.pone.0342167 (PMC12867261; doi:10.1371/journal.pone.0342167)
Supplement: Attachment — Submitted filename: response_to_reviewers_auresp_3.pdf [file pone.0342167.s003.pdf]

Manuscript ID: PONE-D-25-22965R2

Original Article Title: "Performance evaluation of GPU-based parallel sorting algorithms"

To: PLOS One Editor

Re: Response to reviewers

Dear Editor,

Thank you for allowing a resubmission of our manuscript, with an opportunity to address the reviewers' comments.

We are uploading (a) our point-by-point response to the comments (below) (response to reviewers), (b) a marked-up copy of the manuscript (Revised Manuscript with Track Changes), and (c) a clean updated manuscript ("Main Manuscript"*).*

Best regards,

Mohammed Alaa Ala'anzy et al.

Editor Comments

The reviewers' overall opinions are very divergent regarding whether the paper contains a substantial element of novelty. Therefore, it is difficult to clearly identify the novel contribution of this work.

The authors would like to express their sincere gratitude to the Editors for their valuable time and effort, which have greatly contributed to enhancing the quality of our manuscript. Meanwhile, we would like to emphasize that the contribution and novelty are now clearly presented in the abstract and at the end of the introduction section.

Editor, Concern #1: Some of the reviewers' comments suggest possible improvements and scientific relevance for the journal:

Reviewer 6 refers to three recently published papers that introduce new sorting algorithms, which are already mentioned in the literature review and currently deferred to future work. I recommend performing simulations to compare the proposed algorithm with these algorithms, in addition to the classical ones.

Author response: We value the editor's note and have incorporated the improvement accordingly.

Author action: We investigated the recently published paper (2025) called "*SlowSort: An Enhanced Sorting Algorithm for Large Scale Integer Datasets. Software: Practice and Experience.*" and made a full comparison of its performance against QS, BS, MS and RS to find the best algorithm with updated values and graphs. The full description of the Slow Sort (SS) located in **CUDA** section, **Page 12, Line 378** with all formulas. Moreover, the comparison in time complexity with graphs and tables are provided in the **Results and Evaluation** section, **Page 14, Line 470**.

Editor, Concern #2: Moreover, none of the figures (histograms and other visual representations) are in line with the quality standards of the journal. An initial reference figure is missing - a general pipeline or schematic - that could help the reader immediately understand what the work is about.

Author response: We thank the Editor for highlighting the importance of figure clarity and workflow presentation.

Author action: All result figures have been regenerated in high resolution to comply with the journal's quality standards **(Figures 3–5, Page 15).**

In addition, an initial reference schematic has been added as **Figure 1, Page 3** in the Introduction section, illustrating the overall workflow of dataset generation, CPU and GPU sorting algorithms, and performance comparison. This overview figure allows readers to immediately understand the scope and objective of the proposed study.

Reviewers comments

Reviewer #3

In this manuscript, the GPU-based parallelization of mergesort (MS), quicksort (QS), bubble sort (BS) and radix top-k selection sort (RS) are investigated. Also, the performance of these algorithms is evaluated on GPUs utilizing CUDA.

The authors wish to extend their sincere thanks to Reviewer 3 for his/her time and effort, which have played a significant role in improving the quality of our manuscript.

Reviewer #3, Concern: In the revised manuscript, the following comments should be addressed. The main issue is that the references do not fit with the journal's guidelines. In addition, several authors' names in the references are incorrect and need to be corrected. The authors should revise the reference list accordingly.

Author response & action: We thank the reviewer for his observation. We have updated the manuscript by properly checking the references and fixing incorrect names of mentioned authors in **Page 22**. Also, the order and content(author and paper names, dates, etc.) of the reference list was checked carefully and corrected to follow the format and avoid misconnection cases in future.

Reviewer #6

Reviewing the revised version of the paper and authors' responses to the reviewers' comments show that most of concerns are addressed or fixed in the new version of the paper.

The authors wish to extend their sincere appreciation to Reviewer 6 for his/her significant contributions of time and effort, which have substantially enhanced the quality of our manuscript.

Reviewer #6, Concern: One comment of reviewer 6 was about three recently published papers that introduce new sorting algorithms that they are pointed out in the literature review and postponed to the future.

Author response & action: We thank the reviewer for his observation. We examined the recently published 2025 study titled *"SlowSort: An Enhanced Sorting Algorithm for Large Scale Integer Datasets* in *Software: Practice and Experience"* and conducted a comprehensive performance comparison against QS, BS, MS, and RS using updated metrics as time complexity and visualizations such as graphs. A detailed description of the Slow Sort (SS) algorithm, including its CUDA based formulation and associated analytical expressions, is presented in the **CUDA** section on **Page 12, Line 378**. Additionally, comparative analyses of time complexity supported by graphs and tables are provided in the Results and Evaluation section on **Page 14, Line 470**.

We would like to emphasize that some of the suggested papers are not strictly aligned with our work. Accordingly, we have included only the one that is aligned, namely the SlowSort paper.

We thank the reviewers & editors for their thoughtful critiques, which helped us improve the clarity, scope, and scientific rigour of our work.
